# *Plasmodium falciparum* merozoite surface protein 2: epitope mapping and fine specificity of human antibody response against non-polymorphic domains

**DOI:** 10.1186/1475-2875-13-510

**Published:** 2014-12-19

**Authors:** Saidou Balam, Sope Olugbile, Catherine Servis, Mahamadou Diakité, Alba D’Alessandro, Geraldine Frank, Remy Moret, Issa Nebie, Marcel Tanner, Ingrid Felger, Thomas Smith, Andrey V Kajava, François Spertini, Giampietro Corradin

**Affiliations:** Department of Biochemistry, University of Lausanne, Ch des Boveresses 155, 1066 Epalinges, Switzerland; Hospital of St Raphael, 1450 Chapel Street, New Haven, CT 06510 USA; Immunogenomic Units, Malaria Research and Training Centre (MRTC), University of Bamako, Bamako, Mali; Department of Pharmaceutical Sciences of University of Salerno, via Ponte Don Melillo, Fisciano, 84084SA Italy; Hôpital Saint Camille, 01 BP 364, Ouagadougou 01, Burkina Faso; Centre National de Recherche et de Formation sur le Paludisme, 1487 Avenue de l’Oubritenga, BP 2208, Ouagadougou 01, Burkina Faso; Swiss Tropical and Public Health Institute, Socinstrasse 57, 4051 Basel, Switzerland; Centre de Recherches de Biochimie Macromoléculaire, CNRS, Université de Montpellier 1 and 2, 1919 route de Mende, Montpellier, France; University ITMO, 197101 St. Petersburg, Russia; Division of Immunology and Allergy, Centre Hospitalier Universitaire Vaudois (CHUV), 1011 Lausanne, Switzerland; University Hospital Regensburg, Franz-Josef-Strauss-Allee 11, 93053 Regensburg, Germany

**Keywords:** *Plasmodium falciparum*, MSP2, Dimorphic regions, C-terminal region, Epitope mapping, Fine specificity

## Abstract

**Background:**

Two long synthetic peptides representing the dimorphic and constant C-terminal domains of the two allelic families of *Plasmodium falciparum* merozoite surface proteins 2 are considered promising malaria vaccine candidates. The aim of the current study is to characterize the immune response (epitope mapping) in naturally exposed individuals and relate immune responses to the risk of clinical malaria.

**Methods:**

To optimize their construction, the fine specificity of human serum antibodies from donors of different age, sex and living in four distinct endemic regions was determined in ELISA by using overlapping 20 mer peptides covering the two domains. Immune purified antibodies were used in Western blot and immunofluorescence assay to recognize native parasite derivate proteins.

**Results:**

Immunodominant epitopes were characterized, and their distribution was similar irrespective of geographic origin, age group and gender. Acquisition of a 3D7 family and constant region-specific immune response and antibody avidity maturation occur early in life while a longer period is needed for the corresponding FC27 family response. In addition, the antibody response to individual epitopes within the 3D7 family-specific region contributes to protection from malaria infection with different statistical weight. It is also illustrated that affinity-purified antibodies against the dimorphic or constant regions recognized homologous and heterologous parasites in immunofluorescence and homologous and heterologous MSP2 and other polypeptides in Western blot.

**Conclusion:**

Data from this current study may contribute to a development of MSP2 vaccine candidates based on conserved and dimorphic regions thus bypassing the complexity of vaccine development related to the polymorphism of full-length MSP2.

**Electronic supplementary material:**

The online version of this article (doi:10.1186/1475-2875-13-510) contains supplementary material, which is available to authorized users.

## Background

One strategy of malaria intervention is the development of a safe and effective blood-stage vaccine against *Plasmodium falciparum* [1, 2]. The complex life cycle of *P. falciparum*, the wide range of blood stage proteins and their extensive polymorphism provide many options for vaccine design but also render selection of the appropriate antigen(s) and vaccine development challenging. To overcome obstacles to vaccine development resulting from antigen polymorphism, this study focused only on non- or semi-polymorphic regions of promising vaccine candidates [3–7]. One of these is merozoite surface protein 2 (MSP2), a blood-stage protein that appears to be essential for viability and completion of the *Plasmodium* life cycle in humans [8–11]. While its function is not known, it induces specific antibodies (Abs) that are active *in vitro* against parasite merozoites [12–14] and are associated with protection in endemic areas.

MSP2 is a glycosylphosphatidylinositol (GPI)-anchored protein present on the merozoite surface consisting of about 200–250 amino acids, encoded by a single exon on chromosome 2. It contains conserved N- and C-terminal (C) regions flanking a highly polymorphic central repeat region [15]. A non-repeat semi-conserved dimorphic (D) region defines the two allelic families of MSP2: 3D7 and FC27 [16]. D and C region families display low structural complexity due to the high percentage of hydrophilic residues, and are predicted and shown to represent “intrinsically unstructured regions” [4, 17]. It has been shown that specific semi-immune Ab against MSP2 protein is predominantly cytophilic IgG3, as in other blood stage proteins [4, 12, 13, 18]. These cytophilic (IgG1 and IgG3) Abs are thus thought to play an important role in antibody-mediated mechanisms of parasite clearance [19, 20].

A full-length recombinant MSP2 protein was tested in clinical trials as one of the constituents of a three-component malaria vaccine, Combination B [21, 22], containing ring-infected erythrocyte surface antigen (RESA), MSP1 and MSP2 (3D7 variant). The product was safe and partially protective. This effect was, at least in part, due to the immune response against the MSP2-3D7 allele. The 3D7-MSP2 vaccinated group had lower prevalence of parasites carrying this allelic form, while a higher incidence of morbidity episodes was associated with heterologous FC27-type infections [21–24]. These findings suggested that: i) inclusion of both allelic families in a MSP2-based vaccine should increase its efficacy, and ii) an immune response against the highly variable repeat region of MSP2 was probably not involved in protection from 3D7 parasite infection, since the 3D7 repeat present in the vaccine was found very rarely in MSP2 variants in the study area. In a recent phase I clinical trial, a recombinant vaccine candidate containing both the 3D7 and FC27 full-length proteins showed that the majority of vaccinated subjects elicited Abs that were specific for both forms of MSP2 and active in inhibiting parasite growth in antibody-dependent cellular inhibition (ADCI) [25].

In our own investigations only D and C domains of both MSP2 allelic variants were considered, due to the high polymorphism of the central region of MSP2, while the non-polymorphic N-terminal region was excluded because it favours amyloid fibril formation within the MSP2 molecule [26] which potentially leads to regulatory issues. The choice of D and C domains was motivated by the finding showing that the Abs against the 3D7 family specific and constant domain (D + C) was associated with protection from clinical malaria and were active in ADCI [4, 12, 23]. The present study used overlapping 20-mer peptides and donors from different endemic areas, varying by age and sex to identify fragments within the D and C regions with promising vaccine potential.

## Methods

### Synthetic peptides

**Figure 1:**
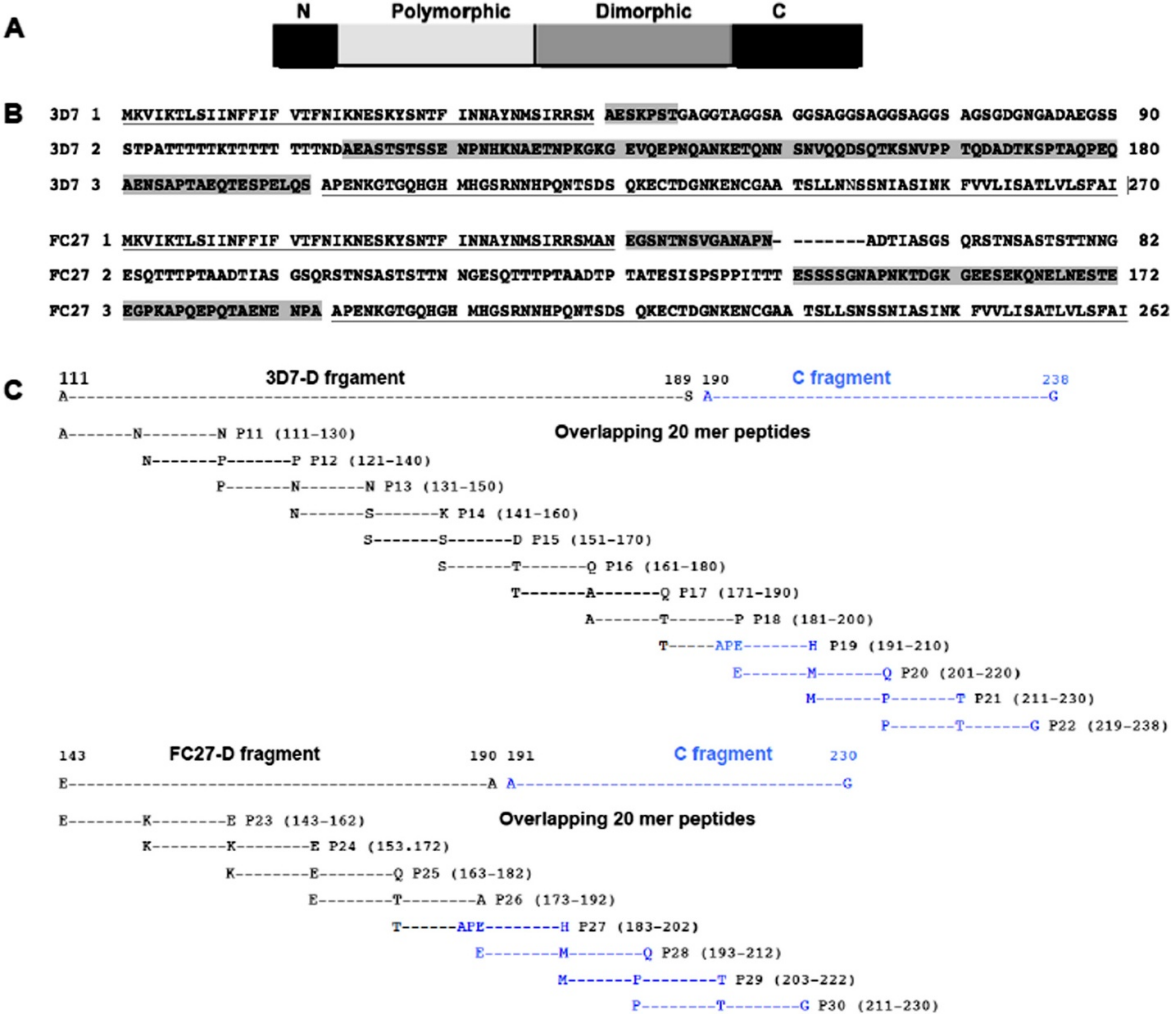
**Schematic structure, full-length alignment sequence, and overlapping 20 mers covering D and C regions of MSP2. A)** Represents schematic structures of merozoite surface protein 2 (MSP2) with different domains (modified from Flueck *et al.*[4]). Mature MSP2 consists about 200–250 amino acids and contains two conserved domains (black, N-and C-termini) flanking a polymorphic region that includes repetitive sequences (light grey) and a non-repetitive dimorphic domain (dark grey). The lengths of the non-conserved domains are strain-dependent. The dimorphic region defines two allelic families of MSP2, 3D7 and FC27. **B)** Shows sequence of the two allelic full-length sequences (from N-to C-terminal) of MSP2 (3D7 and FC27). Non-repeat family-dimorphic regions are shaded in light gray, conserved (N- and C-termini) sequences are underlined. The non-shaded and non-underlined sequences represent the polymorphic regions. **C)** Shows sequence of Dimorphic and constant C-region (blue) sequences, and overlapping 20 mers covering the two regions. In parentheses is the sequence position number. LSP: long synthetic peptide.

The schematic structure and full-length sequence alignment of the two allelic families of MSP2 are shown respectively in Figures 1A and 1B. All peptides were synthesized at the Department of Biochemistry, University of Lausanne, Switzerland, using an Applied Biosystem 431A instrument (Foster City, CA, USA) and Fmoc chemistry and capping of the unreactive peptide chains with acetic anhydride at each cycle. The purity of each peptide (>80%) was determined using analytical C18 HPLC and mass spectroscopy. Lyophilized peptides were dissolved in phosphate buffered saline (PBS (Gibco® Invitrogen™)) at a concentration of 1 mg/ml [4]. Different LSPs derivate from both allelic families of MSP2 are represented in Additional file 1. Overlapping 20-mer peptides (overlapping by ten amino acids) were synthesized by using the MultiRespep Synthesizer (Bioanalytical Instrument, Intavis AG). Thus, 12 20-mer peptides (P11-P22) and eight 20-mer peptides (P23-P30) covered, respectively, 3D7 and FC27-MSP2 D + C region. Thus, the 3D7 20 mers P20, P21 and P22 are similar to the FC27 20 mers P28, P29 and P30. Peptides P19 (from 3D7 family) and P27 (from FC27 family) contained the eight last aa of the D regions, respectively, plus the 12 first aa of their C region (Figure 1C).

### Human plasma

Human sera or plasma samples were collected during the malaria transmission season in each endemic area. Adult donors were from Burkina Faso (BF), Papua New Guinea (PNG), Tanzania (TZ), Nigeria (NG). Donors from Mali were categorized into three age groups (≤five years, six to 14 years and ≥15 years).

For the analysis of association of antibody responses with protection two hundred and eighty sera from children of one to five years of age in the village of Idete in Tanzania were available. These children formed the placebo group of an SPf66 vaccine efficacy trial [27]. All illness episodes in these children reporting to the local health centre during a one-year follow-up after collection of these sera were recorded, and malaria slides prepared for determination of parasite positivity and densities. For analysis of the relationship of protection to antibody levels a clinical malaria episode was defined as measured fever (> or = 37.5 degrees C) and parasite density > 20,000/microL. This was the same definition as was used in the original trial [27]. Research and ethical clearance was granted by the Tanzanian Commission for Science and Technology (UTAFITI NSR/RCA 90).

Sera from Swiss naive donors (12 to 13 individual donors) who had no malaria history were used as a negative control. Blood was taken by venipuncture into tubes containing EDTA according to the ethical clearances of each country. Research carried out on humans was conducted in compliance with the Helsinki Declaration. Some donors with high Ab titres were selected in single or pooled to purify Abs (pAbs) reactive to D, C and D + C fragments of the two allelic families of MSP2, and 3D7-D + 8aaC LSP of 3D7 family.

### Affinity purification of human polyclonal antibodies

Reactive Abs to FC27-D + C, 3D7-D + 8aa C, 3D7-D, FC27-D and C LSPs were purified from ELISA positive plasma from BF, PNG, NG adults and different age groups from Mali using affinity chromatography as previously described [28]. Briefly, 5 mg of peptides were added to 1 ml of activated sepharose and processed according to manufacture instructions.

### Enzyme-linked immunosorbent assay

For enzyme-linked immunosorbent assay (ELISA), antigen concentration for coating 96-well flat plates (BD Biosciences) was 1 μg/ml for peptides longer than 40 residues, and 5 μg/ml for peptides shorter or equal to 40 residues. As previously described [5, 28, 29], plates were coated overnight (O/N) with 50 μl of peptide at the appropriate concentration in PBS and then blocked for one hour at room temperature (RT) in PBS containing 5% non-fat milk powder. Test was considered positive if the sample OD mean was ≥ OD mean + 3 SD of negative controls (non-exposed Swiss donors) or if the ratio of sample OD mean divided by OD mean of negative controls was ≥2. Each sample was tested in duplicate.

### Antibody avidity

A native protein is characterized by its folded structure; increasing the chaotropic characteristics of solvent leads to denaturation. Guanidine chloride (GdCl, Merck KGaA, Darmstadt, Germany) is one of the general protein denaturants leading to protein unfolding [30, 31]. Briefly, for measuring Ab avidity by ELISA, different concentrations of GdCl (8 M, 4 M, 2 M, 1 M and 0 M) were mixed with plasma for 30 min before transferring the mixture into peptide-coated plates. Relative avidity was determined by the concentration of GdCl needed to obtain 50% of the OD value in the absence of GdHCl. Thus samples with OD at 1 M GdHCl higher or equal to 50% of the OD in the absence of GdHCl (0 M) were considered having higher avidity than those with less than 50% of the OD in the absence of GdHCl.

### Immunofluorescence and Western Blot

Immunofluorescence assay (IFA) was performed on blood smear slides containing at least 5% schizontes of 3D7 and FC27 *P. falciparum* cultures and Western blot (WB) were performed using reduced 3D7 and FC27 parasite lysates [5, 28].

### Statistical analysis

Pearson’s Chi-square or Fisher’s exact tests were used to assess association between categorical variables and ANOVA tests (GraphPad Prism, version 5.00) were used to test differences between groups in antibody levels. The relationship between antibody-levels measured in ELISA and the incidence of clinical malaria in the Tanzanian children was evaluated by Kaplan Meier analysis, including tests of significance using log rank chi-square tests. The effects were tested both with age-adjustment, and without adjustment for age.

## Results

### Recognition of individual domains by human sera and delineation of B cell epitopes

**Figure 2:**
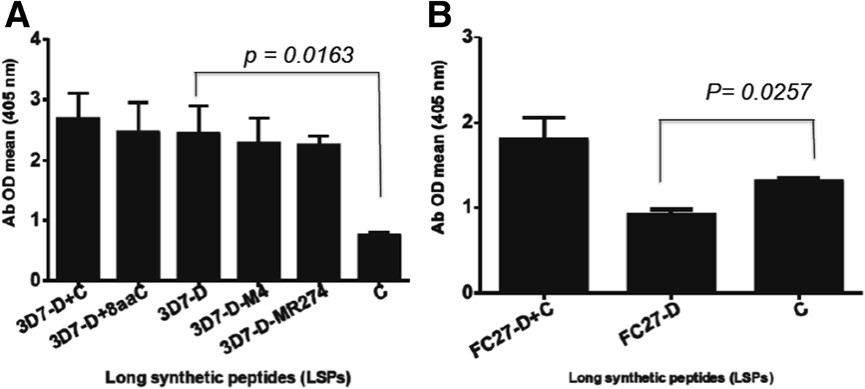
**D + C long synthetic peptides purified antibodies from immune adult recognized D and C.** ELISA was performed using LSPs covering D + C or D and C fragments of the two MSP2 allelic families and purified antibody (pAb) from immune adult pooled sera from Nigerian donors. **A** and **B** represent, respectively, LSP representing 3D7 and FC27 allelic families. The mean of the OD at a dilution 1/100 was determined. **A** and **B** show one-way ANOVA tests comparing the immune response (OD) to the 3D7-D and FC27-D regions to those to the C region respectively.

The intrinsically unstructured D and C offers an ideal opportunity to study the fine specificity of MSP2-reactive human Abs using long and/or short overlapping peptides. The lengths of different LSPs covering D and C regions of the two allelic family regions are showed in Additional file 1. Purified Abs (pAbs) reactive with D + C of each allelic family (obtained from Nigerian adult pooled plasma) and tested in ELISA showed that each dimorphic derivate and C region LSP was well recognized. However, 3D7-D LSP was better recognized than that of C region while the contrary was observed for FC27-D LSP, respectively (Figure 2A and B). Cytophilic IgG1 and IgG3 were the most prevalent subclasses recognizing the 3D7-D and C LSP while recognition of FC27-D LSP was mainly associated with IgG1 (Additional file 2).

In order to study the fine specificity of the antibody response outlined above, 20-mer peptides (overlapping by 10 aa) covering the two allelic regions, D and C regions of MSP2 were tested.

First, sera of adults living in four different endemic regions were tested in ELISA to see if MSP2 20 mers were equally recognized. The prevalence (% positive samples in ELISA test), OD and SD of Ab responses for each peptide are shown in Table 1. The most frequently recognized 20 mers in the 3D7-D region were P13, which was recognized by 67 and 51% of donors from PNG and TZ, respectively, and P13/16, recognized by 46% of the donors from Mali, as well as P17 recognized by 69% from BF. For the FC27-D region, the most prevalent 20 mers were P23 with 48% in PNG, P25 with 45 and 18%, respectively in BF and in Mali, and P26 with 51% in TZ. For the C region, P29 appeared the most prevalent in PNG (50%), BF (39%) and in Mali (13%) while in TZ the most frequent was P30 with 43% (Table 1). Overall, considering the overall prevalence in the four areas, P13 (51%) and P16 (48%) were the most prevalent for the 3D7-D; P23 (28%) and P25 (31%) were for the FC27-D, and P29 and P30 (30%) for C-region (Table 1). However, the frequency of recognition of these commonly seen 20 mers, except for P13 (immunodominant epitopes) differed significantly among the four endemic areas (p <0.05). At all sites, 3D7-D was more frequently recognized than FC27-D or the C region while the frequency was similar among FC27-D and C regions (Table 1).

**Table 1:** **MSP2 D and C domain epitope mapping in different endemic areas**

	Peptides	PNG (N = 42)			Tanzania (N = 37)			BF (N = 49)			Mali (N = 39)			Sum of positves: n (%)	p value
		***Positive: n (%)***	***OD mean***	***SD***	***Positive: n (%)***	***OD mean***	***SD***	***Positive: n (%)***	***OD mean***	***SD***	***Positive: n (%)***	***OD mean***	***SD***		
**3D7 family**	D	41 (98)	2.24	*1.29*	33 (89)	1.47	*1.29*	49 (100)	2.01	0.74	38 (97)	1.75	*1.32*	161 (96)	0.058
	P11	2 (5)	0.28	*0.11*	5 (14)	0.22	*0.09*	13 (27)	0.42	0.69	4 (10)	0.14	*0.07*	24 (14)	**0.011**
	P12	13 (31)	0.55	*0.61*	8 (22)	0.30	*0.25*	16 (33)	0.49	0.53	16 (41)	0.49	*0.91*	53 (32)	0.120
	**P13**	**28 (67)**	0.85	*1.03*	**19 (51)**	0.53	*0.95*	19 (39)	0.61	0.78	**18 (46)**	0.54	*0.91*	**84 (50)**	0.125
	P14	19 (45)	0.64	*0.68*	10 (27)	0.50	*0.83*	28 (57)	1.09	1.25	10 (25)	0.29	*0.43*	67 (40)	**0.003**
	P15	17 (40)	0.60	*0.67*	9 (24)	0.40	*0.60*	22 (45)	0.82	0.95	10 (25)	0.40	*0.60*	58 (35)	0.107
	**P16**	14 (33)	0.60	*0.64*	9 (24)	0.57	*1.19*	27 (55)	0.85	0.84	**18 (46)**	0.60	*0.89*	**68 (41)**	**0.028**
	**P17**	8 (19)	0.30	*0.27*	10 (27)	0.47	*0.92*	**34 (69)**	0.96	0.93	6 (15)	0.24	*0.24*	58 (35)	**<0.001**
	P18	1 (2)	0.24	*0.07*	13 (35)	0.28	*0.23*	14 (29)	0.48	0.68	5 (13)	0.23	*0.50*	33 (20)	**0.001**
	P19	3 (7)	0.22	*0.10*	8 (22)	0.25	*0.23*	3 (6)	0.23	0.17	4 (10)	0.18	*0.24*	18 (11)	0.115
**FC27 family**	D	40 (90)	1.21	*0.82*	31 (84)	1.01	*0.81*	42 (86)	1.47	1.11	25 (65)	0.72	*1.06*	138 (83)	**0.009**
	**P23**	**20 (48)**	0.40	*0.49*	7 (19)	0.23	*0.11*	16 (33)	0.64	0.88	5 ()13	0.28	*0.69*	**48 (29)**	**0.002**
	P24	3 (8)	0.24	*0.22*	3 (8)	0.19	*0.17*	11 (22)	0.32	0.21	4 (10)	0.27	*0.49*	21 (12)	0.114
	**P25**	15 (36)	0.39	*0.58*	9 (24)	0.44	*0.50*	**22 (45)**	0.54	0.63	**7 (18)**	0.17	*0.12*	**53 (32)**	**0.035**
	**P26**	17 (40)	0.32	*0.29*	**11 (30)**	0.36	*0.58*	11 (22)	0.33	0.31	5 (13)	0.16	*0.11*	44 (26)	**0.018**
	P27	3 (8)	0.17	*0.06*	4 (11)	0.27	*0.44*	4 (8)	0.25	0.11	2 (5)	0.17	*0.26*	13 (8)	0.806
**C region**	C	29 (69)	0.57	*0.31*	26 (70)	0.58	*0.62*	33 (67)	0.76	0.67	16 (41)	0.34	*0.31*	104 (62)	0.061
	P28	2 (5)	0.20	*0.10*	0 (0)	0.22	*0.05*	4 (8)	0.23	0.13	1 (2)	0.16	*0.17*	7 (4)	0.384
	**P29**	**21 (50)**	0.40	*0.31*	7 (19)	0.41	*0.56*	**19 (39)**	0.41	0.32	**5 (13)**	0.15	*0.08*	**52 (31)**	**0.001**
	**P30**	16 (38)	0.23	*0.11*	**16 (43)**	0.40	*0.64*	13 (27)	0.39	0.39	**5 (13)**	0.19	*0.17*	**50 (30)**	**0.017**

Table 1 Showed ELISA performed on LSPs and 20 mers covering the D and C fragments of the two allelic families of MSP2. Adult plasma from PNG (N = 42), TZ (N = 37), BF (N = 49) and Mali (N = 39) were obtained during malaria season transmission. ELISA was considered positive if the ratio of mean OD of test sample/mean OD of negative control was more than or equal to 2. Peptides in bold indicate the major recognized 20 mers. Means of Ab OD, standard deviation (SD), and p-values from Chi-square tests (comparing the proportion of responders among the four endemic areas for each peptide) were determined. P values ≤ 0.05 are in bold and considered statically significant.

These data showed that all 20-mer peptides were antigenic and led to the delineation of immunodominant MSP2 epitopes in endemic areas tested.

### Fine specificity of D and C region recognition in different age groups

Epitope mapping was extended to donors divided into three age groups (children ≤ five years, adolescents six to 14 years and adults ≥15 years) living in Mali. Samples were collected during the malaria transmission season. The prevalence of the most recognized 20 mers of the 3D7-D and C domains did not depend on the age of donors (Table 2). In contrast, for the FC27-D region, acquisition of immune responses against the 20 mers increased significantly with age, in particular, there were significant differences between the children and adults children (Table 2).

**Table 2:** **MSP2 D and C domains epitope mapping in different age groups**

	Peptides	Children (1) (≤5 years) N = 38			Adolescents (2) (6–14 years) N = 43			Adults (3) (≥15 years) N = 26			p value		
		***Positive: n (%)***	***Mean OD***	***SD***	***Positive: n (%)***	***Mean OD***	***SD***	***Positive: n (%)***	***Mean OD***	***SD***			
											1 v 2	1 v 3	2 v 3
**3D7 family**	D	32 (84)	1.26	0.97	43 (100)	1.50	1.35	26 (100)	2.05	0.94	**0.008**	**0.008**	1
	P11	0 (0)	0.11	0.01	2 (5)	0.12	0.02	8 (31)	0.17	0.11	0.148	**0.001**	**0.007**
	P12	8 (21)	0.20	0.09	18 (42)	0.66	0.94	8 (31)	0.41	0.37	**0.038**	0.386	0.346
	P13	16 (42)	0.64	0.95	20 (47)	0.44	0.73	11 (42)	0.40	0.37	0.690	0.987	0.733
	P14	11 (29)	0.16	0.07	12 (28)	0.18	0.13	13 (50)	0.51	0.66	0.918	0.086	0.065
	P15	13 (34)	0.18	0.13	24 (56)	0.39	0.53	9 (35)	0.59	0.83	**0.045**	0.973	0.078
	P16	13 (34)	0.43	0.54	19 (44)	0.67	0.93	12 (46)	0.53	0.70	0.356	0.337	0.874
	P17	5 (13)	0.20	0.16	22 (51)	0.21	0.16	7 (27)	0.29	0.35	**<0.0001**	0.181	**0.036**
	P18	2 (5)	0.14	0.08	2 (5)	0.13	0.02	6 (23)	0.33	0.56	0.899	**0.048**	**0.038**
	P19	14 (37)	0.22	0.31	4 (9)	0.11	0.01	2 (8)	0.20	0.27	**0.002**	**0.002**	0.814
**FC27 family**	D	23 (61)	0.21	0.11	37 (86)	0.85	1.15	23 (88)	0.76	0.50	**0.007**	**0.006**	0.768
	P23	2 (5)	0.11	0.02	12 (28)	0.50	0.94	9 (35)	0.15	0.06	**0.003**	**0.003**	0.562
	P24	0 (0)	0.14	0.04	4 (9)	0.45	0.83	7 (27)	0.21	0.13	**0.036**	**0.002**	0.071
	P25	4 (11)	0.13	0.05	7 (16)	0.20	0.17	10 (38)	0.19	0.12	0.444	**0.009**	**0.045**
	P26	4 (11)	0.11	0.02	10 (23)	0.18	0.17	8 (31)	0.17	0.08	0.118	**0.050**	0.499
	P27	6 (16)	0.21	0.36	6 (14)	0.11	0.02	2 (8)	0.20	0.29	0.817	0.305	0.400
**C region**	C	14 (37)	0.27	0.28	19 (44)	0.25	0.12	14 (54)	0.50	0.40	0.500	0.175	0.435
	P28	6 (16)	0.17	0.21	2 (5)	0.13	0.02	3 (23)	0.19	0.22	0.098	0.473	**0.038**
	P29	5 (13)	0.12	0.04	11 (26)	0.13	0.04	8 (31)	0.18	0.11	0.150	0.096	0.644
	P30	2 (5)	0.13	0.05	5 (12)	0.13	0.03	7 (27)	0.28	0.26	0.296	**0.022**	0.125

Table 2 showed ELISA performed on LSPs and 20 mers covering the D and C fragments of the two allelic families of MSP2 using sera from age groups living in Mali. Plasma was used at dilution 1/200. The ELISA was considered positive if the ratio of mean Ab OD of test sample/mean OD of negative control was more than or equal to 2. Ab OD, SD and p-values from Fisher’s exact test (comparison of the proportions positive between two age groups) were calculated for each peptide. Numbers (1, 2, 3) represent respectively children, adolescents and adults code. v: *versus.* P values ≤ 0.05 are in bold and considered statically significant.

**Figure 3:**
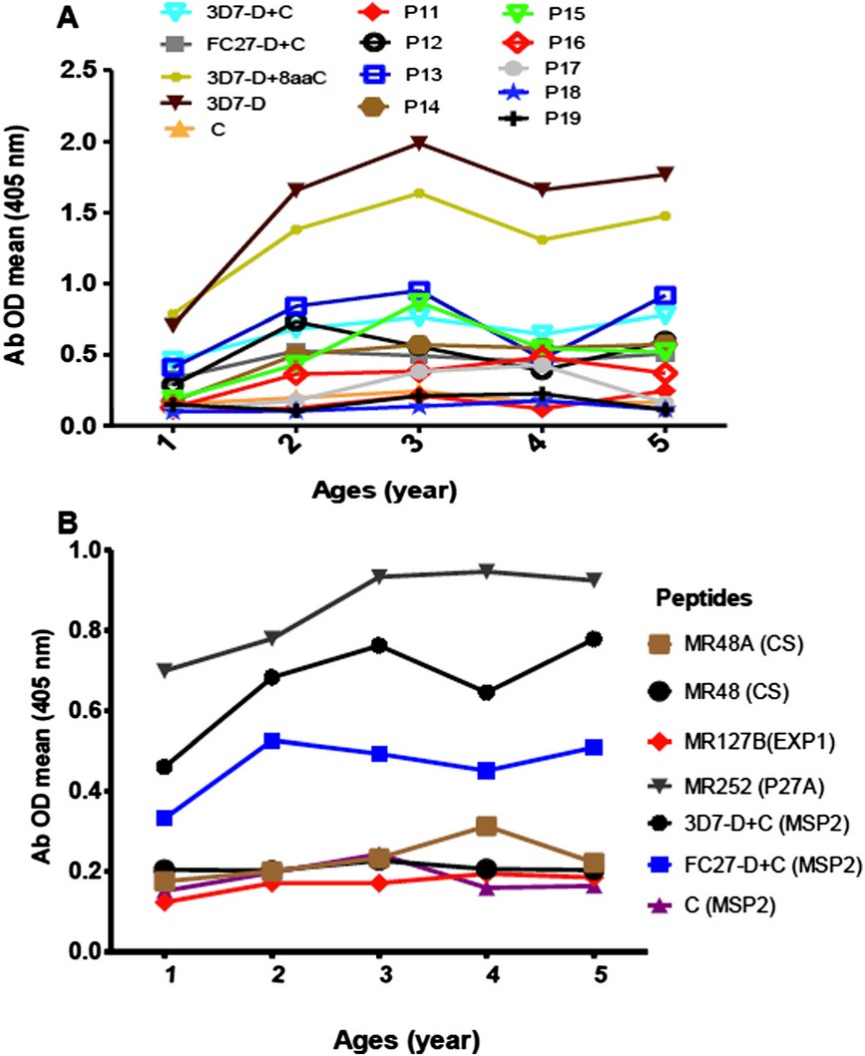
**Acquisition of immune response against D and C regions of MSP2 occurs early in life in malaria-exposed donors.** Sera from children of different ages (1–5 years old) living in Tanzania were collected and used in direct ELISA. **A)** Shows age dependent recognition of 3D7-D and its 20 mers peptides (N: number total of children = 186). **B)** Shows age dependent recognition of different LSPs representing P27A (MR252; N = 208), exported protein 1, EXP1 (MR127B; N = 254) and CS (MR48A, MR48; N = 254). Sera were used at dilution of 1/200.

In the context of this evidence that immune responses against the D and C regions are acquired early in individuals exposed to malaria, the detailed age profile of immune response acquisition in the earlier life of donors was examined in the 186 sera from Tanzanian children of different ages (one to five years). Immune responses against most of peptides increased with age until two to three years old, but tended to decrease in four years old children. Thereafter Ab responses remained stable or increased again (Figure 3A). A similar pattern of Ab responses was observed also for LSPs covering D + C region of the two MSP2 allelic families, the C region alone (Figure 3B). To determine if this was a general phenomenon, other unstructured LSPs derived from a blood or pre-erythrocytic stage proteins, PFF0165c, P27A, MR252 [5], exported proteins *Pf*EXP1, MR127B [32, 33], and N-terminal circumsporozoite (CS) protein fragments (MR48 and MR48A [34]) (Additional file 1) were tested in ELISA. Interestingly, only Ab responses against LSPs from MSP2 increased with age until two to three years followed by a modest decrease in four years old while those to EXP1, CS and P27A LSPs tended to increase or remain stable at this age (Figure 3B).

### Relative avidity of antibodies to D- and C-LSPs and their immunodominant epitopes in different age groups

Antibody avidity is one of the parameters used to determine the maturation of an immune response [35–39]. Relative avidity of Abs reactive to different fragments of MSP2 and their 20 mers was determined by using different concentrations of GdCl at a fixed serum dilution (1:200) in ELISA (data not shown). Eleven plasma samples per age group from Malian donors that gave the highest OD in ELISA against D and C were tested. By considering the OD value curve at different concentrations of the GdCl (i.e., the number of samples with OD at 1 M GdCl higher than 50% of the OD at no GdCl), the relative avidity between the 3D7-D LSP and its specific Ab appeared similar for different age groups as shown in Additional file 3A. In contrast, relative avidity for FC27-D specific Abs was low in children and increased with age (children < adolescents < adults, (children *versus* adults, p <0.05 (*)) (Additional file 3B)) whereas, relative avidity of Abs for the C region was higher in children compared to adolescents and adults (p <0.05 (*) and P ≤ 0.001 (**), respectively) (Additional file 3C). These results are consistent with those obtained above regarding age dependency of immune responses.

### Association of the immune antibody response elicited against individual 3D7-D 20 mers in children with protection from malaria

**Figure 4:**
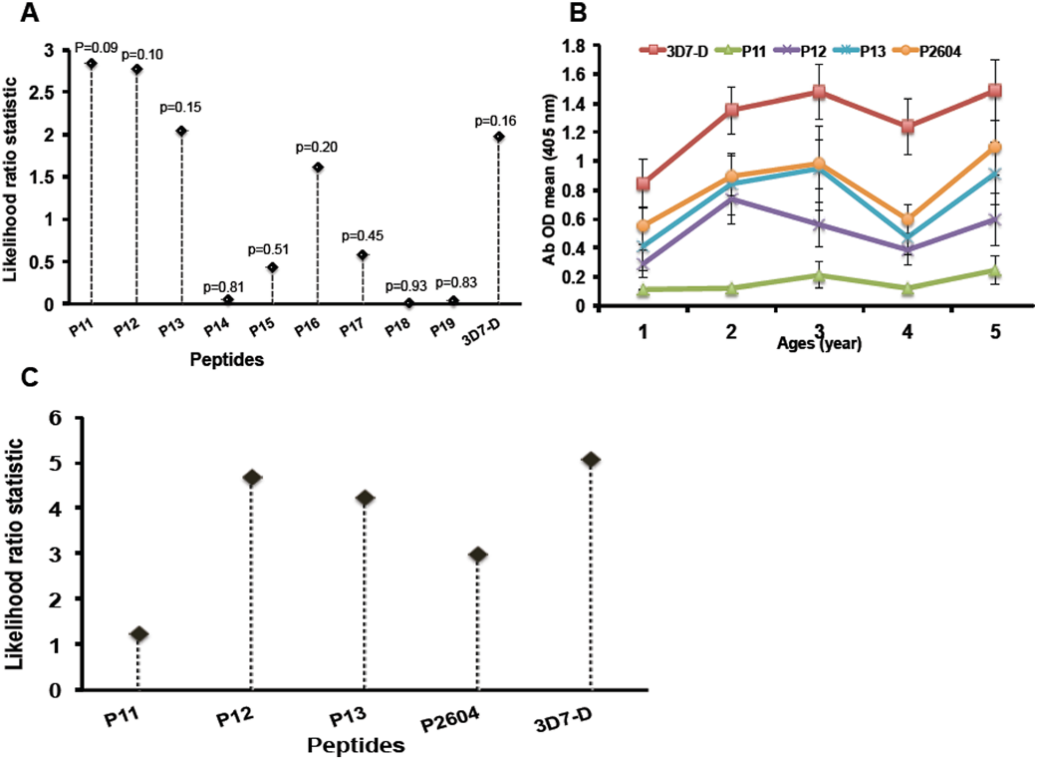
**Association of antibody response against 3D7-D 20 mers and protection from malaria in children.** Sera from children 1–5 years old (N = 186) living in Tanzania were used at dilution 1/200 to perform ELISA on 3D7-D LSP and its corresponding 20 mers. **A)** Log rank chi-squares statistics from Kaplan-Meier analysis testing the association of malaria morbidity rates with adjusted Ab OD for age. The p values (p) test the hypothesis that the 3D7-D and each 20-mer specific antibodies are associated with clinical protection. Since peptides P11, P12 and P13 showed the highest likelihood association values with protection, P2604 containing those three 20 mers sequences was synthesized and tested in children 1–5 years old (N = 190) living in Tanzania with its constitutive 20 mers, 3D7-D and 3D7D - + 8aaC. **B)** Shows profile curve of Ab responses (arithmetic mean OD). **C)** Shows Log rank Chi-square from Kaplan-Meier analysis determining the relationship between malaria morbidity, Ab OD and age for each peptide.

It has been shown earlier [4] that the antibody response to 3D7 D + C but not FC27 D + C region is associated with protection from malaria clinical malaria. In addition, the antibody response to 3D7-D + 8aa C-terminal conserved residues was also associated with protection in the Tanzanian children cohort. This analysis was then extended to 3D7-D LSP and its corresponding 20 mers in the same Tanzanian children. The relationship between malaria morbidity and antibody OD at baseline was determined for each peptide. Peptides P11, P12, P13 and P16 were associated with the highest probability in protection (Figure 4A). Except for P11, the peptides P12, P13 and P16 were among the immunodominant epitopes of 3D7-D as shown above in Table 1. These epitopes showed a slightly increased or similar association with protection compared to the reference LSP of 3D7-D. However, their association with protection was not statistically significant (p >0.05) (Figure 4A).Since peptides P11, P12 and P13 showed the strongest association with protection, a longer peptide, called P2604 (111–150; 40 aa) containing the three sequences was synthesized. ELISA test performed in the cohort of Tanzanian children indeed showed an increased recognition P2604, but the association with protection did not reach a significance level (Figure 4B and C) for either peptide P2604 or D.

### Cross-reaction of D- and C-LSP purified antibodies in WB and IFA

**Figure 5:**
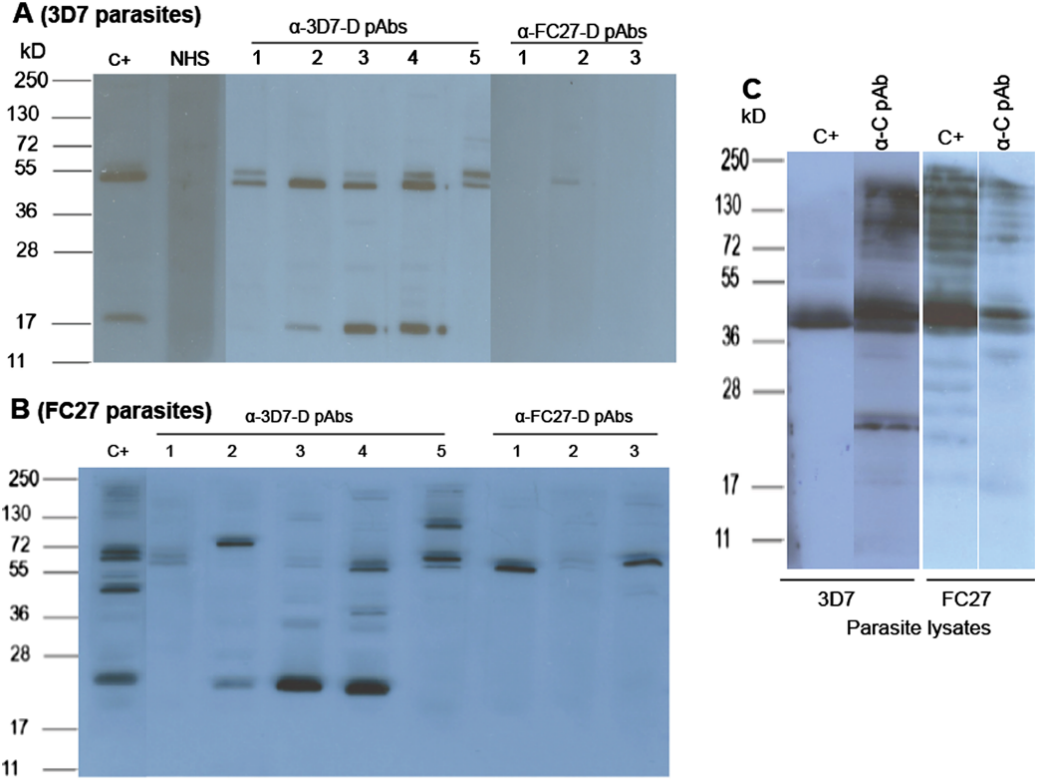
**D and C affinity purified antibodies recognized polypeptides from the two allelic family of MSP2.** Western Blot (WB) was performed using 3D7 and FC27 merozoites. **A** and **B** show respectively, specific 3D7- and FC27 D-individual adult pAbs (Burkina Faso, BF) against MSP2 allelic-derived polypeptides in WB, used at dilution of 1/100. Naive human sera (NHS) and C+ (pAb from 3D7-D + 8aa **C)** from Nigerian adult pool plasma were used as negative and positive controls, respectively. **C)** The C-terminal reactive-pAb from individual BF donor (that gave highest Ab titre in ELISA) was obtained to perform WB on both MSP2 allelic family parasites at a dilution of 1/100.

As reported previously [14], 3D7-D and FC27-D human reactive Abs inhibited heterologous parasite growth as determined in ADCI and recognized heterologous MSP2 recombinant proteins in ELISA. To confirm these findings, analysis was extended using 3D7-D, FC27-D and C LSP affinity purified Abs from single adult BF donors. In WB, the FC27-D pAbs recognized only family-specific proteins whereas 3D7-D pAbs recognized both 3D7 and FC27 *P. falciparum*-derived proteins (Figure 5A and B). Cross-reactive bands observed were depending on serum donor (Figure 5A). As expected, the C region-reactive pAbs recognized both 3D7 and FC27-strain derived merozoite proteins (Figure 5C). Note that MSP2 is localized between 55–35 kD included. Additionally, pAbs from plasma pools of donors of different ages and single adult C-reactive pAbs were used to perform IFA on both of the 3D7 and FC27 schizonts. All of the 3D7-D specific pAbs recognized both homologous (3D7) and heterologous (FC27) merozoites confirming the cross-binding reaction (Additional file 4A and B). Due to the low level of antibody titre against the FC27-D region found in children and adolescents, the reverse experiment could not be performed. As for WB, C region-reactive pAbs recognized both 3D7 and FC27 parasites in IFA (Additional file 4C).

## Discussion

The aim of the current studies was the delineation of immunodominant and protective D and C epitopes in individuals living in different malaria-endemic areas in order to develop more effective MSP2-based universal vaccines either as a single candidate or in combination with other antigens.

Here, the first observation was that the immunodominant 20-mer epitopes contained in each single MSP2 domain (P13, P15/16 for 3D7-D; P23, P25/P26 for FC27 and P29/P30 for C) are in part conserved in the four endemic areas analysed (Table 1). Differences in the level and specificity of serum or affinity purified antibodies of single individuals are observed and may depend on the history of infections by different *P. falciparum* variant strains and/or on the genetic diversity of donors [16, 40, 41]. In general, recognition of the 3D7-D major epitopes is more prevalent than that observed for FC27-D and C regions (Table 1) in agreement with data shown by other groups either for the full-length proteins or fragments thereof [3, 4, 7]. In addition, Ab responses against D or C region of each MSP2 allelic family and individual 20 mers were similar in male and female populations living in the same endemic area (Additional file 5), thus facilitating the development of universal MSP2-based vaccines.

The acquisition of an optimal response as judged by ELISA is obtained early in life (age two to three years) for the 3D7-D and its 20 mers as seen for several pre-erythrocytic and erythrocytic antigens (Figure 3A, B). This is further confirmed when the relative avidity of the Ab response in the three age groups was determined. In contrast, for the FC27-D region, as observed for the magnitude of the response, the apparent avidity of Ab is low in the children, and increases with age (Additional file 3). Together, these data confirm that the response to 3D7 MSP2 matures earlier in life compared to that to FC27 MSP2, most likely due to a higher rate of infection by 3D7-family parasites [7, 42, 43]. In addition, the relative avidity of Abs against C region appeared strongest in children compared to that shown in adolescent and adult groups (Additional file 3). This confirms observations from other groups who showed that the positive Ab responses to C-region were observed only in children and not in adults [44–46].

Antibodies to *P. falciparum* MSP2 antigens belong predominantly to the cytophilic and complement-fixing subclasses IgG1 and IgG3, and have been shown to be associated with protection from clinical malaria in immune adults [13, 47, 48]. This study confirmed that IgG1 and IgG3 are the most relevant subclasses associated with MSP2 LSP recognition in adults, but also in adolescent and children (Additional file 2).

As described before [10, 14, 49, 50], 3D7- and FC27-D pAbs recognize the heterologous RecMSP2 (recombinant MSP2 protein) in ELISA and here parasite-derived proteins in WB and IFA (Figure 5, Additional file 4). The cross-binding between the two allelic MSP2 families occurred only between MSP2 full-length recombinant proteins or parasite-derived proteins but not for LSP. Moreover, the cross binding was strictly dependent on the donors, suggesting a possible influence of parasite infections experienced by single donors and/or individual genetic differences. Cross-binding with higher molecular weight protein(s) was also observed using mouse sera or monoclonal antibodies derived by mouse immunization with MSP2 peptides [50]. Bio-informatic analysis indicates a number of possible cross-reacting proteins (Additional file 6), and proteomic experiments are needed to identify them. As expected, C region specific pAbs recognized both allelic parasite-derived proteins, further justifying its inclusion in a MSP2 vaccine development. The extensive cross-reaction discussed above may be beneficial for the acquisition of a protective immune response.

Finally, the association of the Abs response to 3D7-D region and its epitopes with protection from clinical malaria was not statistically significant (p >0.05) but showed a diverse degree of probability, with P11, P12 and P13 mers having the highest probability. Analysis of the fragment P2604 containing the P11, P12 and P13 sequences led to increased recognition by Ab in different age groups (compared to single constitutive 20 mers) but was not associated with protection. Similarly, repeat testing of the same cohort for the antigen D +8aaC gave smaller estimates of relative risk, suggesting that the promising result previously obtained may well be a chance finding. Thus, testing of other paediatric cohorts is needed to assess the impact of D + 8aaC in protection.

## Conclusion

Previous [4, 14, 50] and present data further support the notion that family-specific and constant regions of MSP2 are promising vaccine candidates, which should bypass the limitations of using the full-length, highly polymorphic MSP2 proteins as vaccines.

## Electronic supplementary material

Additional file 1: **Sequences of different long synthetic peptides.** The bold and underlined sequences correspond to the common C-terminal region sequence of MSP2-3D7 and FC27 families. 3D7 dimorphic derivate LSPs are 3D7-D (88 aa), 3D7-D-M4 (73 aa) and 3D7-D-MR274 (60 aa). D + C is sequence derivate from dimorphic of each family plus the C region. Note that M and MR plus different number represent only peptide code given during synthesis. Sequences of LSPs derivate from ***Pf*** **EXP1 (MR127B)**, **PFF0165C (MR252 or P27A)** and *P. falciparum* circumsporozoite: *Pf*CS **(MR48, MR48A from N-terminal)** are also represented. (DOC 40 KB)

Additional file 2: **Isotyping of IgG against D and C domain LSPs of the two allelic families of MSP2.** D and C fragments of the two MSP2 allelic families were used to perform ELISA in order to define the subclass of total IgG. Individual plasma samples from children (N = 12), adolescents (N = 13), and adults (N = 14) living in Mali were used at a dilution of 1/200. ELISA was considered positive if mean of Ab OD of test sample with considered IgG subtype was more than mean OD + 3SD of negative control sera (N: 12). Percentage of positive sera for IgG1, IgG2, IgG3 and IgG4 against each region of MSP2 was thus determined. (PDF 266 KB)

Additional file 3: **Determination of relative age-specific antibody avidity against D and C regions.** Plasma from different age groups (children, adolescents and adults from Mali, 11 sample per age group) that elicited high Ab responses in ELISA were mixed with different concentrations of GdCl (0 to 8 M) for 30–45 minutes before transfer to ELISA plates containing corresponding peptide. A, B and C represent respectively, the relative Ab avidity to 3D7-D, FC27-D and C region LSPs. The p values show statistic difference (Fisher’s exact test) between two age groups. ns: not statistically significant, *: p < 0.05 and **: p ≤ 0.001. (PDF 98 KB)

Additional file 4: **3D7-D and C LSP purified antibodies recognized merozoite proteins derived from the two allelic family parasites.** Immunofluorescence staining of malaria-infected erythrocytes was performed with age specific 3D7-D (A, B) respectively y, 3D7 and FC27 strain merozoites, and C-terminal reactive pAbs (C). The age reactive pAbs specific to 3D7-D and C were obtained from age-pooled plasma (A and B), respectively and single BF plasma (C), and used at a dilution of 1/100. Nucleus stained with DAPI (in blue) and transmission picture of the infected red blood cell (DIC). (PDF 296 KB)

Additional file 5: **D and C epitope prevalence of the two allelic MSP2 according to the gender of donors.** Plasma of Malian donors (regardless of age): females (F, N = 57) and males (M, N = 50) were collected during the malaria season transmission and used at dilution 1/200 to perform direct ELISA on 20 mers covering the two domains of the two MSP2 allelic families. Sample was considered positive responder if ratio of mean Ab OD/mean OD of negative control was equal to or more than 2. The p value was calculated from Fisher’s exact test that compares variation between the two sexes for each peptide (n = number;%: percent of positive donors). N = total number of donor from each sex. (DOC 66 KB)

Additional file 6: **Similar shared sequences between the two allelic families of MSP2.** Similar possible and shared sequences are presented here in order to explain the cross-binding occurred between the two allelic families. For this purpose, dimorphic sequences of each MSP2 allelic family were matched with those representing the heterologous family. A and B represent, respectively, sequences of FC27 and 3D7 dimorphic matched with their own and heterologous sequences, whereas C shows matching of common C-terminal sequences with the two allelic regions (minimal epitope is four amino acids). Upper case letter indicates the same amino acids; lower case letter represents the different amino acids. All data were generated from PlasmoDB database [51]. (DOC 204 KB)
