# Supplementary material for: Plasmodium falciparum merozoite surface protein 2: epitope mapping and fine specificity of human antibody response against non-polymorphic domains
Source: Malar J. 2014 Dec 19;13:510. doi: 10.1186/1475-2875-13-510 (PMC4320585; doi:10.1186/1475-2875-13-510)
Supplement: Supplementary file 1 — Additional file 1: Sequences of different long synthetic peptides. The bold and underlined sequences correspond to the common C-terminal region sequence of MSP2-3D7 and FC27 families. 3D7 dimorphic derivate LSPs are 3D7-D (88 aa), 3D7-D-M4 (73 aa) and 3D7-D-MR274 (60 aa). D + C is sequence derivate from dimorphic of each family plus the C region. Note that M and MR plus different number represent only peptide code given during synthesis. Sequences of LSPs derivate from Pf EXP1 (MR127B), PFF0165C (MR252 or P27A) and P. falciparum circumsporozoite: PfCS (MR48, MR48A from N-terminal) are also represented. (DOC 40 KB) [file 12936_2014_3667_MOESM1_ESM.doc]

|  | **LSPs** | **Proteins** | **Sequences** | **Number of aa** | **N° of sequences** |
| --- | --- | --- | --- | --- | --- |
| **3D7-D+C** | | ***Pf*3D7-MSP2** | AEASTSTSSENPNHKNAETNPKGKGEVQEPNQANKETQNNSNVQQDSQTKSNVPPTQDADTKSPTAQPEQAENSAPTAEQTESPELQS**APENKGTGQHGHMHGSRNNHPQNTSDSQKECTDGNKENCG** | *128* | *111-238* |
| **3D7-D+8aaC** | | ***Pf*3D7-MSP2** | AEASTSTSSENPNHKNAETNPKGKGEVQEPNQANKETQNNSNVQQDSQTKSNVPPTQDADTKSPTAQPEQAENSAPTAEQTESPELQS**APENKGTG** | *96* | *111-206* |
| **3D7-D** | | ***Pf*3D7-MSP2** | AEASTSTSSENPNHKNAETNPKGKGEVQEPNQANKETQNNSNVQQDSQTKSNVPPTQDDTKSPTAQPEQAENSAPTAEQTESPELQS | *88* | *111-198* |
| **3D7-D-M4** | | ***Pf*3D7-MSP2** | NAETNPKGKGEVQEPNQANKETQNNSNVQQDSQTKSNVPPTQDADTKSPTAQPEQAENSAPTAEQTESPELQS | *73* | *126-198* |
| **3D7-D-MR274** | | ***Pf*3D7-MSP2** | PNQANKETQNNSNVQQDSQTKSNVPPTQDADTKSPTAQPEQAENSAPTAEQTESPELQS | *60* | *140-198* |
| **FC27-D+C** | | ***Pf*FC27-MSP2** | ESSSSGNAPNKTDGKGEESEKQNELNESTEEGPKAPQEPQTAENENPA**APENKGTGQHGHMHGSRNNHPQNTSDSQKECTDGNKENCG** | *88* | *143-230* |
| **FC27-D** | | ***Pf*FC27-MSP2** | ESSSSGNAPNKTDGKGEESEKQNELNESTEEGPKAPQEPQTAENENPA | *48* | *143-191* |
| **C** | | **3D7/FC27-MSP2** | **APENKGTGQHGHMHGSRNNHPQNTSDSQKECTDGNKENCG** | *40* | *198-238* |
| **MR252 (P27A)** | | **PFF0165C** | HNNNEKNISYDKNLVKQENDNKDEARGNDNMCGNYDIHNERGEMLDKGKSYSGDEKINTSDNAKSCSGDEKVITSDNGKSYDYVKNESEEQEEKENMLNNKKRS | 104 | 223-326 |
| **MR127B** | | ***Pf*EXP1 (liver)** | LYNTEKGRHPFKIGSSDPADNANPDADSESNGEPNADPQVTAQDVTPEQPQGDDNNLVSGPEHA | *64* | *101-163* |
| **MR48** | | ***Pf*CS N-term**  **(NF54 strain)** | EYQCYGSSSNTRVLNELNYDNAGTNLYNELEMNYYGKQENWYSLKKNSRSLGENDDGNNEDNEKLRKPKHKKLKQPADGNPDPNANPNV | *89* | *22-110* |
| **MR48A** | | ***Pf*CS N-term**  **(NF54 strain)** | LKKNSRSLGENDDGNNEDNEKLRKPKHKKLKQPADGNPDPNANPNV | *46* | *65-110* |
