# Supplementary figures and images for: Plasmodium falciparum merozoite surface protein 2: epitope mapping and fine specificity of human antibody response against non-polymorphic domains
Source: Malar J. 2014 Dec 19;13:510. doi: 10.1186/1475-2875-13-510 (PMC4320585; doi:10.1186/1475-2875-13-510)

Number of positives

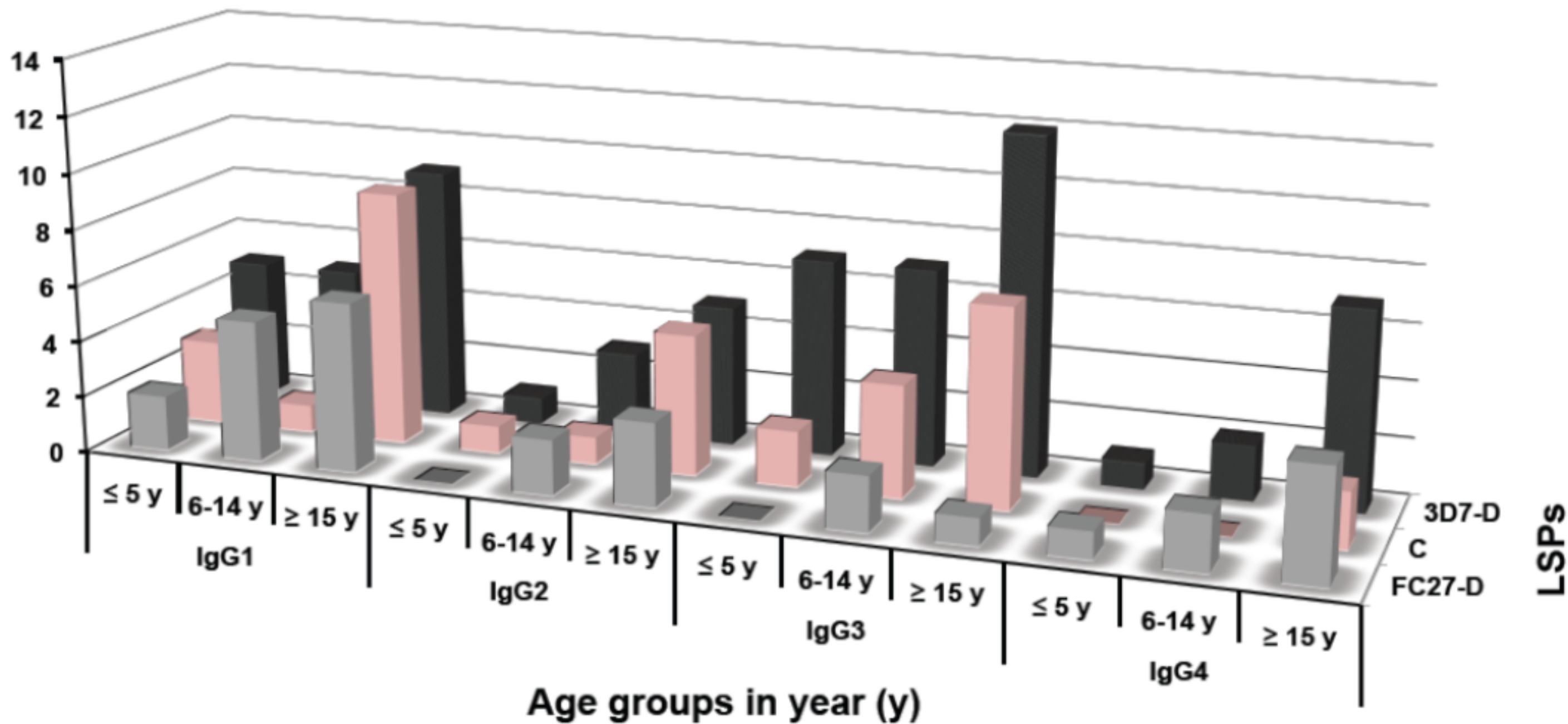

Supplement: Supplementary file 2 — Additional file 2: Isotyping of IgG against D and C domain LSPs of the two allelic families of MSP2. D and C fragments of the two MSP2 allelic families were used to perform ELISA in order to define the subclass of total IgG. Individual plasma samples from children (N = 12), adolescents (N = 13), and adults (N = 14) living in Mali were used at a dilution of 1/200. ELISA was considered positive if mean of Ab OD of test sample with considered IgG subtype was more than mean OD + 3SD of negative control sera (N: 12). Percentage of positive sera for IgG1, IgG2, IgG3 and IgG4 against each region of MSP2 was thus determined. (PDF 266 KB) [file 12936_2014_3667_MOESM2_ESM.pdf]

**A (3D7-D LSP)**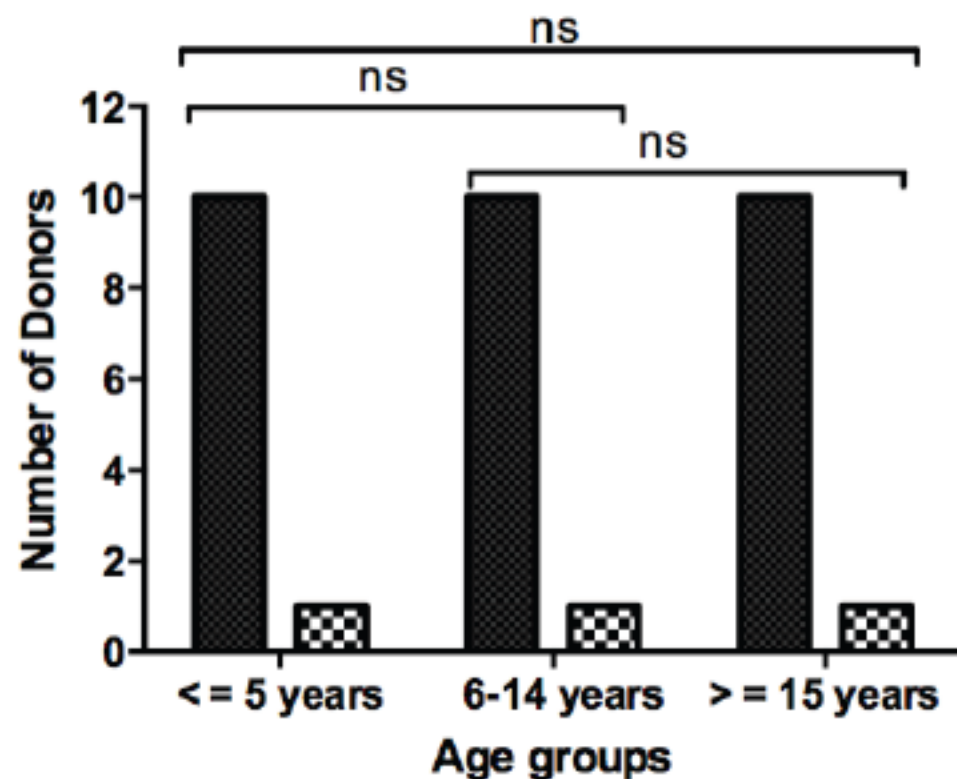**B (FC27-D LSP)**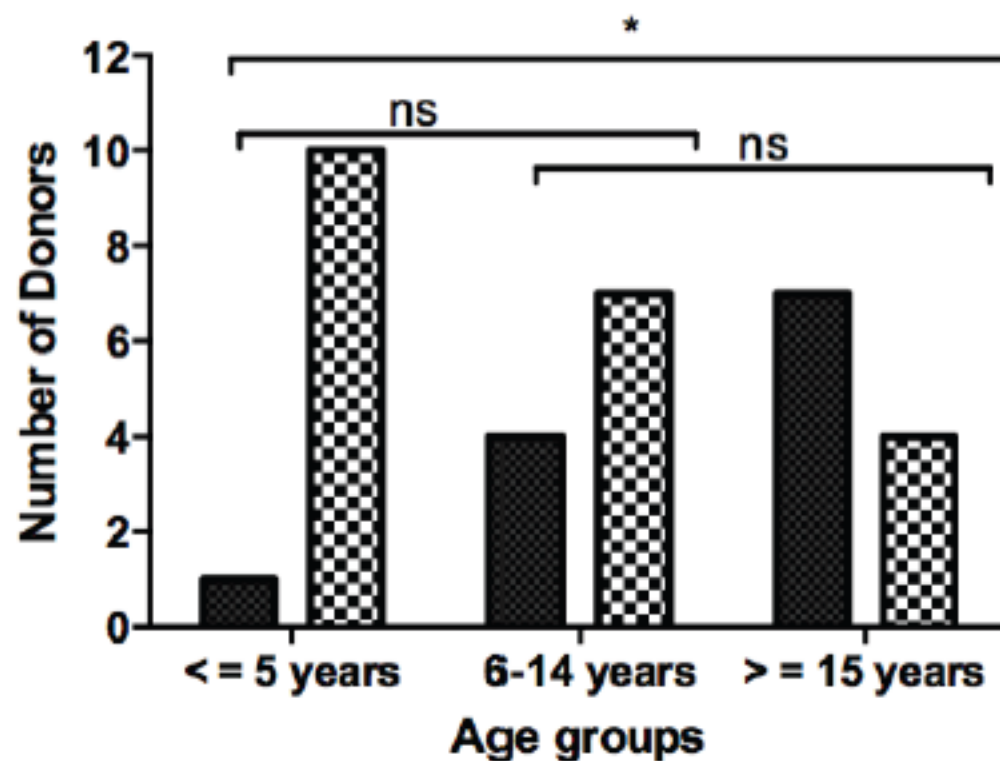**C (C LSP)**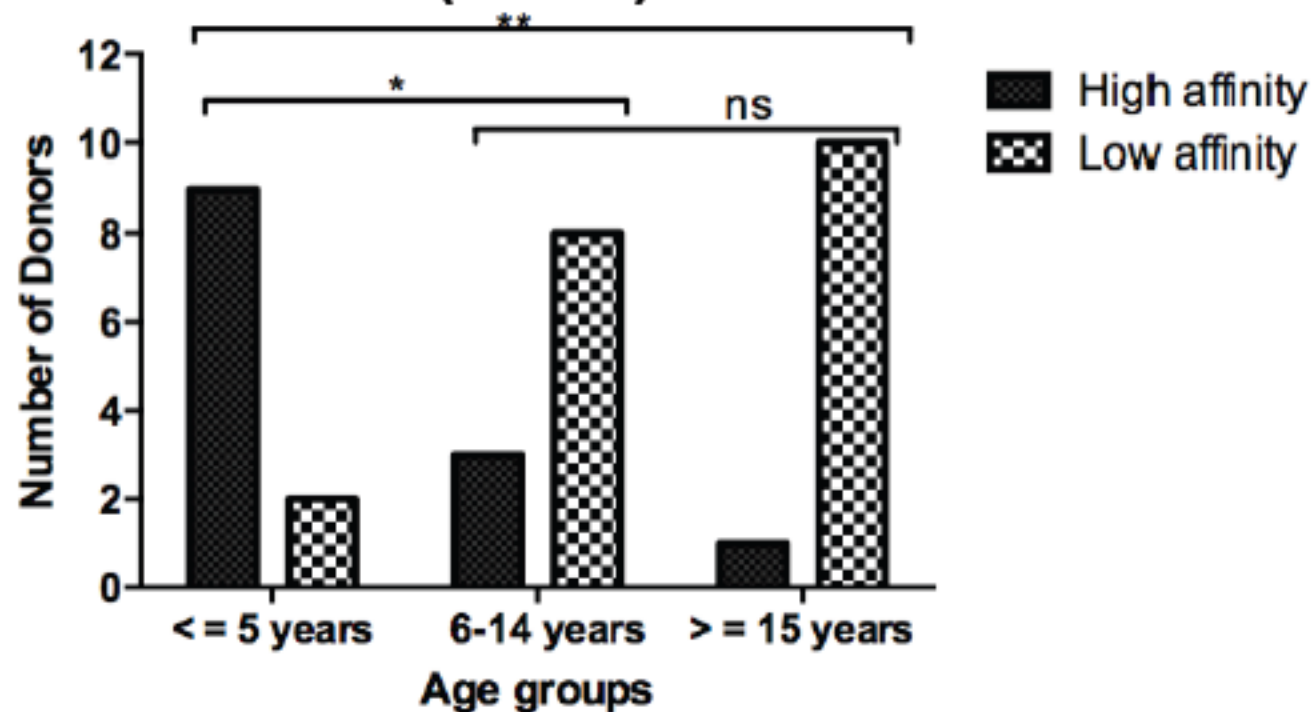

Supplement: Supplementary file 3 — Additional file 3: Determination of relative age-specific antibody avidity against D and C regions. Plasma from different age groups (children, adolescents and adults from Mali, 11 sample per age group) that elicited high Ab responses in ELISA were mixed with different concentrations of GdCl (0 to 8 M) for 30–45 minutes before transfer to ELISA plates containing corresponding peptide. A, B and C represent respectively, the relative Ab avidity to 3D7-D, FC27-D and C region LSPs. The p values show statistic difference (Fisher’s exact test) between two age groups. ns: not statistically significant, *: p < 0.05 and **: p ≤ 0.001. (PDF 98 KB) [file 12936_2014_3667_MOESM3_ESM.pdf]

## A (3D7 parasite)

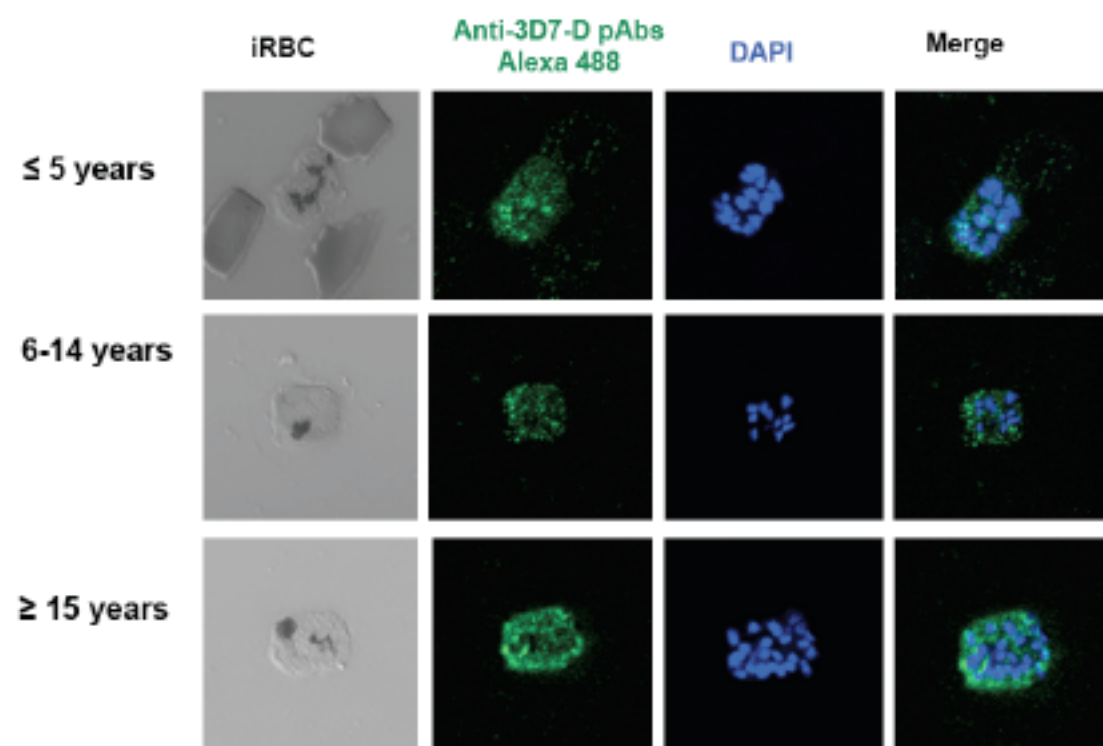

## B (FC27 parasite)

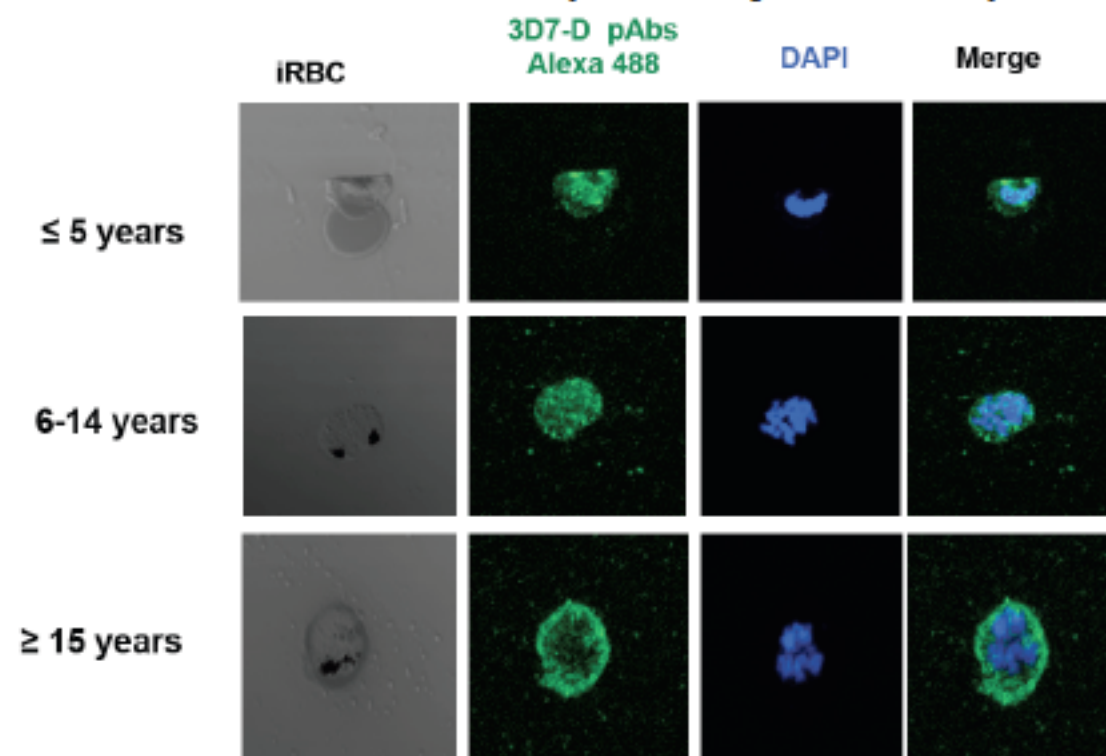

## C

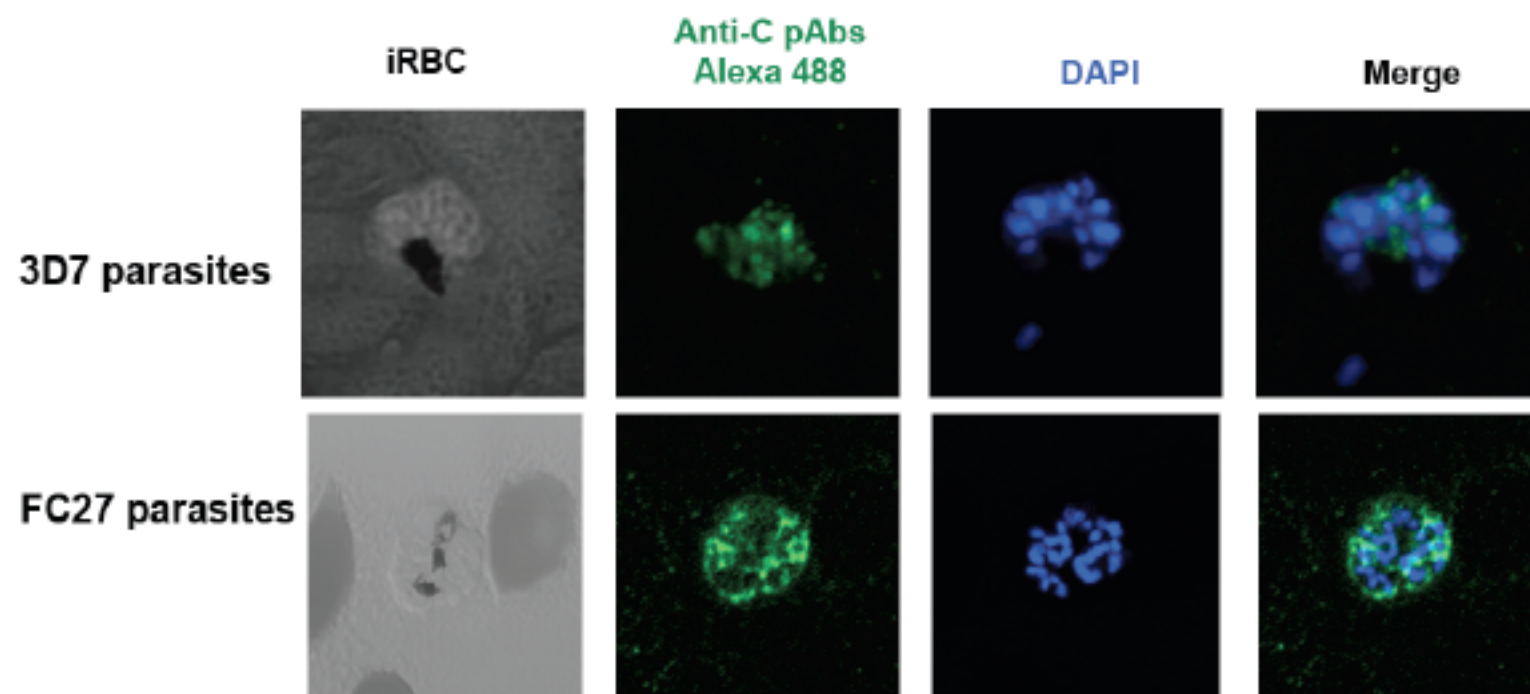

Supplement: Supplementary file 4 — Additional file 4: 3D7-D and C LSP purified antibodies recognized merozoite proteins derived from the two allelic family parasites. Immunofluorescence staining of malaria-infected erythrocytes was performed with age specific 3D7-D (A, B) respectively y, 3D7 and FC27 strain merozoites, and C-terminal reactive pAbs (C). The age reactive pAbs specific to 3D7-D and C were obtained from age-pooled plasma (A and B), respectively and single BF plasma (C), and used at a dilution of 1/100. Nucleus stained with DAPI (in blue) and transmission picture of the infected red blood cell (DIC). (PDF 296 KB) [file 12936_2014_3667_MOESM4_ESM.pdf]
