# Supplementary material for: Plasmodium falciparum merozoite surface protein 2: epitope mapping and fine specificity of human antibody response against non-polymorphic domains
Source: Malar J. 2014 Dec 19;13:510. doi: 10.1186/1475-2875-13-510 (PMC4320585; doi:10.1186/1475-2875-13-510)
Supplement: Supplementary file 5 — Additional file 5: D and C epitope prevalence of the two allelic MSP2 according to the gender of donors. Plasma of Malian donors (regardless of age): females (F, N = 57) and males (M, N = 50) were collected during the malaria season transmission and used at dilution 1/200 to perform direct ELISA on 20 mers covering the two domains of the two MSP2 allelic families. Sample was considered positive responder if ratio of mean Ab OD/mean OD of negative control was equal to or more than 2. The p value was calculated from Fisher’s exact test that compares variation between the two sexes for each peptide (n = number;%: percent of positive donors). N = total number of donor from each sex. (DOC 66 KB) [file 12936_2014_3667_MOESM5_ESM.doc]

|  | **Peptides** | **Female (N=57)** | | | **Male (N=50)** | | | **p value** |
| --- | --- | --- | --- | --- | --- | --- | --- | --- |
|  |  | **Positive: n (%)** | **OD mean** | **SD** | **Positive: n (%)** | **OD mean** | **SD** |  |
|  |  |  |  |  |  |  |  |  |
| **3D7 family** | D | 56 (98) | 1.38 | 0.88 | 45 (90) | 1.56 | 1.07 | 0.072 |
|  | P11 | 5 (8) | 0.29 | 0.41 | 5 (10) | 0.23 | 0.33 | 0.828 |
|  | P12 | 22 (38) | 0.53 | 0.58 | 12 (24) | 0.46 | 0.58 | 0.099 |
|  | P13 | 24 (42) | 0.57 | 0.66 | 23 (46) | 0.52 | 0.64 | 0.685 |
|  | P14 | 18 (31) | 0.42 | 0.57 | 18 (36) | 0.47 | 0.63 | 0.630 |
|  | P15 | 24 (42) | 0.66 | 0.78 | 22 (44) | 0.69 | 0.77 | 0.843 |
|  | P16 | 22 (38) | 0.64 | 0.78 | 22 (44) | 0.63 | 0.71 | 0.571 |
|  | P17 | 18 (31) | 0.55 | 0.70 | 16 (32) | 0.43 | 0.56 | 0.963 |
|  | P18 | 4 (7) | 0.23 | 0.28 | 6 (12) | 0.24 | 0.35 | 0.383 |
|  | P19 | 13 (22) | 0.33 | 0.34 | 7 (14) | 0.29 | 0.28 | 0.235 |
| **FC27 family** | D | 46 (80) | 0.67 | 0.52 | 37 (74) | 0.70 | 0.75 | 0.409 |
|  | P23 | 14 (24) | 0.36 | 0.46 | 9 (18) | 0.31 | 0.47 | 0.405 |
|  | P24 | 6 (10) | 0.26 | 0.39 | 5 (10) | 0.26 | 0.32 | 0.929 |
|  | P25 | 10 (17) | 0.30 | 0.28 | 11 (22) | 0.32 | 0.35 | 0.564 |
|  | P26 | 13 (22) | 0.29 | 0.20 | 9 (18) | 0.26 | 0.15 | 0.536 |
|  | P27 | 10 (17) | 0.30 | 0.24 | 4 (8) | 0.28 | 0.24 | 0.132 |
| **C region** | C | 24 (42) | 0.46 | 0.39 | 23 (46) | 0.44 | 0.41 | 0.685 |
|  | P28 | 5 (8) | 0.26 | 0.14 | 9 (18) | 0.29 | 0.25 | 0.162 |
|  | P29 | 13 (22) | 0.35 | 0.29 | 11 (22) | 0.28 | 0.22 | 0.920 |
|  | P30 | 9 (15) | 0.33 | 0.34 | 5 (10) | 0.24 | 0.12 | 0.368 |
