# Supplementary material for: Plasmodium falciparum merozoite surface protein 2: epitope mapping and fine specificity of human antibody response against non-polymorphic domains
Source: Malar J. 2014 Dec 19;13:510. doi: 10.1186/1475-2875-13-510 (PMC4320585; doi:10.1186/1475-2875-13-510)
Supplement: Supplementary file 6 — Additional file 6: Similar shared sequences between the two allelic families of MSP2. Similar possible and shared sequences are presented here in order to explain the cross-binding occurred between the two allelic families. For this purpose, dimorphic sequences of each MSP2 allelic family were matched with those representing the heterologous family. A and B represent, respectively, sequences of FC27 and 3D7 dimorphic matched with their own and heterologous sequences, whereas C shows matching of common C-terminal sequences with the two allelic regions (minimal epitope is four amino acids). Upper case letter indicates the same amino acids; lower case letter represents the different amino acids. All data were generated from PlasmoDB database [51]. (DOC 204 KB) [file 12936_2014_3667_MOESM6_ESM.doc]

**A- MSP2-FC27-D specific sequence:**

**ESSSSGNAPNKTDGKEGESEKQNELNESTEEGPKAPQEPQTAENENPA**

**Additional file 6 (continued)**

**B- MSP2-3D7-D specific fragment:**

**AEASTSTSSENPNHKNAETNPKGKGEVQEPNQANKETQNNSNQQDSQTKSNVPPTQDAD KSPTAQPEQAENSAPTAEQTESPELQS**

**Additional file 6 (continued and end)**

**C- Superfamily shared region (C-terminal):**

**APENKGTGQHGHMHGSRNNHPQNTSDSQKECTDGNKENCG**
